# FGF21 alleviates microvascular damage following limb ischemia/reperfusion injury by TFEB-mediated autophagy enhancement and anti-oxidative response

**DOI:** 10.1038/s41392-022-01172-y

**Published:** 2022-10-12

**Authors:** Fanfeng Chen, Jiayu Zhan, Xiaoqing Yan, Abdullah Al Mamun, Yu Zhang, Yitie Xu, Hongyu Zhang, Xiaokun Li, Kailiang Zhou, Jian Xiao

**Affiliations:** 1 Oujiang Laboratory (Zhejiang Lab for Regenerative Medicine, Vision and Brain Health), Department of Vascular Surgery, The First Affiliated Hospital of Wenzhou Medical University, Wenzhou, China; 2 Molecular Pharmacology Research Center, School of Pharmaceutical Science, Wenzhou Medical University, Wenzhou, China; 3 Department of Orthopaedics, The Second Affiliated Hospital and Yuying Children’s Hospital of Wenzhou Medical University, Wenzhou, China

**Keywords:** Cardiovascular diseases, Outcomes research

**Dear Editor**,

Microvascular damage is a pivotal pathological factor in lower limb ischemia/reperfusion (I/R) injury.^1^ Excessive reactive oxygen species (ROS) formation and disrupted autophagic flux have been recognized as the critical mechanism of cellular death,^2,3^ especially in I/R injury. Recent investigation has displayed that fibroblast growth factor 21 (FGF21) exerts a protective effect against I/R injury via transcription factor EB (TFEB)-mediated autophagy and regulation of anti-oxidative response.^4^ However, the promising role of FGF21 in acute lower limb I/R injury remains elusive.

We found that the expression of FGF21 was remarkably elevated after reperfusion and then decreased to initial levels in the mouse model of hind limb I/R (Supplementary Fig. S1a, b). Blood flow signals, measured by laser doppler imaging (LDI), were significantly decreased after reperfusion and the impaired blood flow in the limb persisted even for 72 h (Supplementary Fig. S1c, d). ROS levels that measured by DHE staining were increased immediately after reperfusion and then decreased to initial levels (Supplementary Fig. S1e, f). Combined with these results, FGF21 gene expression may be induced upon injury stimulus after reperfusion. We next investigated whether FGF21 deficiency intensifies I/R damage of limbs in the mouse model. After I/R injury, hypoperfusion of limb, tissue edema and skeletal muscle fiber injury were further deteriorated in FGF21-KO mice compared with wild type (WT) control groups (Fig. 1a–c). Intriguingly, we observed that the microvascular density was further reduced in FGF21-KO mice than in WT groups (Fig. 1d). Those results indicated that FGF21-deficiency aggravated hypoperfusion, skeletal muscle fiber injury, edema and microvascular endothelial damage in I/R limbs. Then, we administered exogenous FGF21 to investigate its potential therapeutic effects. FGF21 administration markedly reversed I/R-induced hypoperfusion, microvascular damage and skeletal muscle injury (Fig. 1e–h). Moreover, FGF21 enhanced the blood perfusion in limbs after 6 h of reperfusion, indicated that FGF21 also play a protective role in the initial stage of I/R injury (Supplementary Fig. S2a, b). Therefore, FGF21 could be a physiological supporter against limb I/R injury and associated vascular and muscle lesions.

**Fig. 1:**
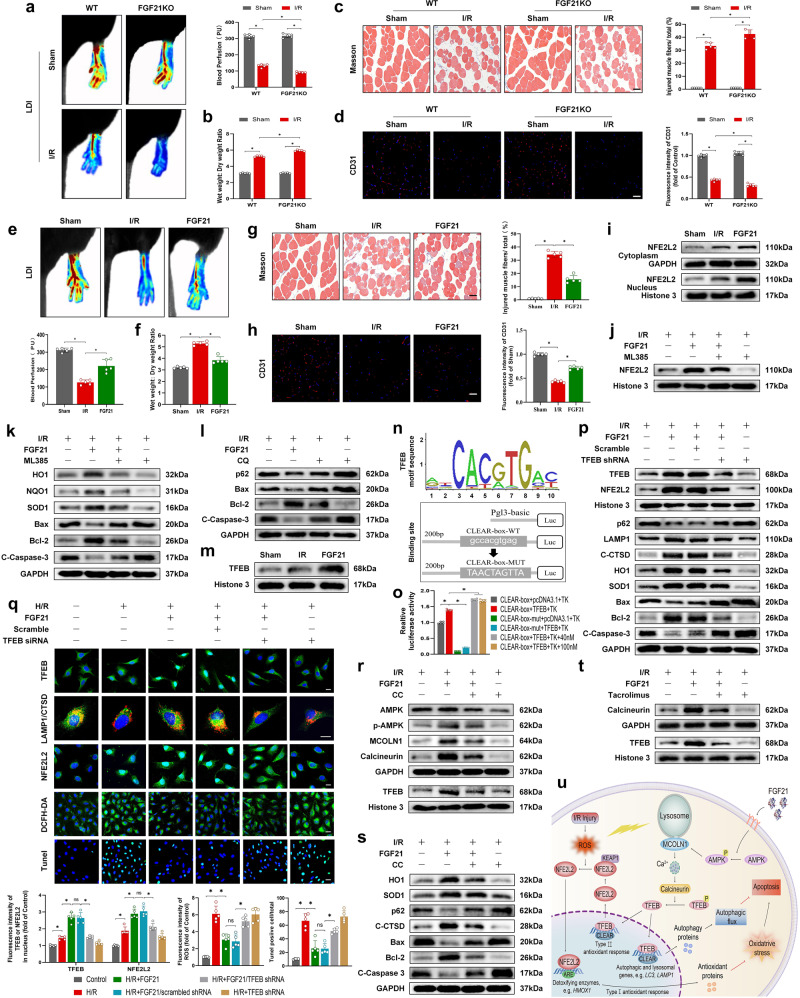
FGF21 protects microvascular ECs against I/R injury by TFEB-mediated autophagy restoration and anti-oxidative response. **a** Left: blood perfusion of FGF21-KO and WT-mice before and after I/R injury were detected by laser doppler imaging (LDI). Right: histogram showing signal intensity of blood flow in I/R limbs. **b** Wet weight to dry weight ratio. **c** Left: masson staining of the transverse sections of skeletal muscle. Scale bar, 50 µm. Right: the degree of skeletal muscle fiber injury was assessed by the percentage of injured fibers. **d** Left: images of skeletal muscle sections from the indicated groups stained with antibodies against CD31; scale bar: 100 µm. Right: quantification of mean optical density values of CD31. **e** Upper: blood perfusion of hind limbs was detected by LDI. Lower: signal intensity of blood flow was plotted as a histogram. **f** Wet weight to dry weight ratio. **g** Left: masson staining of the transverse sections of skeletal muscle. Scale bar, 50 µm. Right: the degree of skeletal muscle fiber injury was assessed by the percentage of injured fibers. **h** Left: images of skeletal muscle sections from the indicated groups stained with antibodies against CD31; scale bar: 100 µm. Right: quantification of mean optical density values of CD31. **i** Western blots for NFE2L2 in the cytoplasm and nucleus in skeletal muscle tissues after FGF21 administration. **j** Western blots for NFE2L2 in the nucleus in skeletal muscle tissues after ML385 administration. **k** Western blots for oxidative stress and apoptosis biomarkers in skeletal muscle tissues after ML385 administration. **l** Western blots for autophagy flux and apoptosis biomarkers in skeletal muscle tissues after CQ administration. **m** Western blots for TFEB in the nucleus in skeletal muscle tissues after FGF21 administration. **n** TFEB motif sequence and TFEB-binding site in CTSD promotor. **o** Quantification of CTSD promotor activity measured with a dual-luciferase assay. **p** Western blots for autophagy flux, oxidative stress and apoptosis biomarkers in skeletal muscle tissues after AAV-TFEB shRNA administration. **q** Upper: representative immunofluorescence images of HUVECs depicting TFEB (scale bars: 25 μm), CTSD and LAMP1 (scale bars: 10 μm) and NFE2L2 (scale bars: 25 μm); and DCFH-DA assay and TUNEL assay of HUVECs (scale bars: 25 μm). Lower: quantification of immunofluorescence intensity displaying the average optical density of TFEB, NFE2L2 and ROS; and the percentages of TUNEL-positive cells versus total cells. **r** Western blotting showing the cytoplasmic levels of AMPK, p-AMPK, MCOLN1 and calcineurin which were corrected by GAPDH as internal control; and nuclear levels of TFEB which were corrected by Histone-3 as internal control after CC administration. **s** Western blots for oxidative stress, autophagy flux and apoptosis markers in skeletal muscle tissues after CC administration. **t** Western blotting showing the cytoplasmic levels of calcineurin which were corrected by GAPDH as internal control; and nuclear levels of TFEB which were corrected by Histone-3 as internal control after tacrolimus administration. **u** Graphic abstract was presented. All the data were shown as mean ± SD (*n* = 4–6 per group). Significance: ns, non-significant; ^*^P < 0.05

Differentially expressed genes (DEGs) were shown by a heatmap (Supplementary Fig. S2e, f). We observed that FGF21 played an influential role in the expression of 2795 genes, in which 2543 genes were considerably upregulated and 252 genes were downregulated. Specifically, gene ontology (GO) analyses revealed that these DEGs were mainly enriched in autophagy, oxidative stress (OS) and apoptosis (Supplementary Fig. S2g). We speculated that FGF21 protects I/R limbs via facilitating autophagic activities and suppressing OS and apoptosis. Therefore, we further assessed the OS, autophagy and apoptosis-related markers in FGF21-KO mice. Compared with WT control groups, the levels of OS-related markers such as HO1, NQO1 and SOD1 were significantly downregulated in FGF21-KO mice (Supplementary Fig. S3a, d). Moreover, FGF21 deficiency aggravated I/R-induced reduction in autophagy flux, as indicated by lower levels of LAMP1 and CTSD, and a higher level of p62 (Supplementary Fig. S3b, e). FGF21 deficiency also aggravated I/R-induced increase in apoptosis level, as indicated by higher levels of Bax and cleaved caspase-3 (CC3), and a lower Bcl-2 level (Supplementary Fig. S3c, f). We then performed a rescue assay to further determine the relationship between FGF21 and apoptosis in the I/R limbs. Z-DEVD-FMK (a specific caspase-3 inhibitor) partially repressed the adverse effects of FGF21 deficiency in blood flow in limbs and the microvascular density in the skeletal muscle (Supplementary Fig. S3i–l), indicated that FGF21 exerts a protective effect by inhibiting apoptosis in I/R limbs. Then, we assessed the changes in OS, autophagy and apoptosis after exogenous FGF21 administration.

The α-SMA (vascular smooth muscle cell maker) signals were not considerably impacted by FGF21 deficiency or exogenous FGF21 (Supplementary Figs. S2c, d, S3g, h), while FGF21 signifcantly rescued the loss of CD31 (endothelial cell marker) signals induced by I/R injury (Fig. 1h). Therefore, FGF21 play a role in endothelial cells (ECs) rather than smooth muscle cells. Based on these findings, HUVECs were selected as the carrier for cell experiments in this study. Our results showed that FGF21 intervention significantly suppressed the levels of Bax and CC3 and increased the levels of Bcl-2 both in skeletal muscle following I/R (Supplementary Fig. S4a, b) and in hypoxia/reoxygenation (H/R)-induced HUVECs (Supplementary Fig. S4e, f). Besides, decreased TUNEL-positive signals on the vascular wall (Supplementary Fig. S4c, d) and in HUVECs (Supplementary Fig. S4g, h) were observed in the FGF21 group. Therefore, FGF21 suppresses I/R-induced apoptosis in ECs.

We also analyzed whether FGF21 inhibits the activation of OS signals. We found that FGF21 remarkably augmented the expression level of HO1, NQO1 and SOD1 proteins in vivo and in vitro (Supplementary Fig. S5a–f). ROS levels were decreased in the FGF21 group in H/R-induced HUVECs (Supplementary Fig. S5g, h). Therefore, FGF21 effectively attenuates OS in I/R-induced ECs. However, the exact pharmacological impacts of FGF21 restricting OS are still elusive in ECs. Nuclear factor, erythroid 2 like 2 (NFE2L2) is a master anti-oxidative transcription factor that regulates OS. We found that FGF21 significantly augmented the expression level of NFE2L2 protein in the cytoplasm and nuclei in vivo (Fig. 1i and Supplementary Fig. S6a, b) and in vitro (Supplementary Fig. S7a–c). The results revealed that FGF21 enhanced the activity of NFE2L2. Then, ML385, a novel small molecule inhibitor of NFE2L2, reversed the anti-oxidative and anti-apoptotic effects of FGF21 on ECs in vivo (Fig. 1j, k and Supplementary Fig. S6c–l) and in vitro (Supplementary Figs. S6m–r, S7a–f). ML385 also reversed the protective effect of FGF21 on limb perfusion and muscle edema after I/R injury (Supplementary Fig. S6s–u). Collectively, these findings clarify that the protection effects of FGF21 on the I/R limbs might be partially due to the removal of excessive ROS via the NFE2L2-mediated anti-oxidative effect.

We further assessed the changes in autophagy after FGF21 administration. Our results indicated that FGF21 markedly elevated Beclin1, LC3, CTSD and LAMP1 levels and decreased p62 protein levels in vivo (Supplementary Fig. S8a, b, d–g) and in vitro (Supplementary Fig. S8o, p). Immunofluorescence results showed that the punctate fluorescence feature of LAMP1 and CTSD and their co-localization in the merged picture, which were decreased in H/R-treated HUVECs, were significantly enhanced upon FGF21 administration (Supplementary Fig. S8q–s). However, the qPCR analyses revealed that p62 levels were not considerably upregulated during I/R, whereas I/R stimulation slightly increased Ctsd mRNA level and FGF21 elevated the gene expression (Supplementary Fig. S8c). Collectively, those findings reveal that FGF21 enhances the activity of autophagy initiation and restores the blocked autophagy flux. We next co-administered FGF21 with chloroquine (CQ), which restrains lysosomal proteases or downstream autophagosome-lysosome fusion and determine whether treatment with FGF21 protects limbs from I/R injury via restoring autophagic flux. Western blot (WB) assay revealed that CQ remarkably elevated LC3II levels in the Sham and FGF21 group. Nevertheless, CQ could not elevate LC3II levels in the I/R group (Supplementary Fig. S8j, k), indicating that the capacity for further autophagosome formation was lacking and the degradation capacity of lysosomes was already at a low level in the I/R group. WB and immunofluorescence results showed that CQ partially reversed the effects of FGF21 on p62 and apoptosis in ECs (Fig. 1l and Supplementary Fig. S8f–i). Furthermore, CQ also reversed the protective effect of FGF21 on limb perfusion and muscle edema after I/R injury (Supplementary Fig. S8l–n). Those findings suggest that FGF21 replenishes autophagy flux via improving lysosomal function in ECs.

To explore the precise effects of FGF21 on autophagy flux and OS posterior to I/R, we further analyzed whether FGF21 plays a modulatory role in TFEB. WB and immunofluorescence results showed that FGF21 markedly enhanced TFEB translocation in vivo (Fig. 1m and Supplementary Figs. S9a, S10a, f) and in vitro (Fig. 1q). Moreover, the dual-luciferase assay results unraveled that FGF21 remarkably reinforced TFEB transcriptional activity as indicated by elevated luciferase activity driven by the CTSD promotor (Fig. 1n, o). Therefore, FGF21 could effectively upregulate the activity of TFEB. We further applied the interfering RNA technique to downregulate TFEB in vivo and in vitro. WB, qPCR and immunofluorescence results revealed that silencing of TFEB abolished the FGF21-induced autophagy restoration, anti-oxidative response and anti-apoptotic effects on ECs both in vivo (Fig. 1p and Supplementary Figs. S9b–f, S10a–j) and in vitro (Fig. 1q). Sulforaphane (SFN, a NFE2L2 agonist) was used to activate NFE2L2, and the results showed the AAV-TFEB shRNA injection reduced the antioxidant capacity and increased the apoptosis level, while the SFN administration partially reversed the adverse effects of the AAV-TFEB shRNA injection (Supplementary Fig. S9j, k). This rescue experiment supports that FGF21 enhances the NFE2L2-mediated anti-oxidative and anti-apoptotic effects partially through activation of TFEB in I/R limbs. Surprisingly, TFEB-gene silencing reversed the protective effect of FGF21 on limb perfusion and muscle edema after I/R injury (Supplementary Fig. S9g–i). Those results imply that FGF21 exerts its therapeutic benefit via enhancing anti-oxidative response and autophagy through the induction of TFEB nuclear translocation.

We next explored the underlying molecular mechanism of FGF21 more clearly. In Supplementary Fig. S2g, GO analyses revealed that calcium ion binding was regulated upward in the FGF21 group. Previous research has shown a vital calcium signal path that modulating the activities of TFEB: the AMPK-mucolipin 1 (MCOLN1)-calcineurin signal transmission cascade.^5^ RNA-seq outcomes revealed that the expression of MCOLN1 and calcineurin in the FGF21 group was remarkably increased (Supplementary Fig. S11a). According to our results, FGF21 activated p-AMPK in the cytoplasm, while no remarkable difference was observed in the expression of AMPK between I/R and FGF21 groups (Supplementary Fig. S11b, c). As downstream signaling molecules, the expression levels of MCOLN1 and calcineurin were remarkably promoted in the FGF21-treated group (Supplementary Fig. S11b, c). Therefore, FGF21 facilitates the AMPK-MCOLN1-calcineurin pathway. To further prove whether the AMPK-MCOLN1-calcineurin signaling pathway modulates FGF21-induced TFEB activation, we assessed the pharmacological impact of CC (a specific inhibitor of AMPK) and tacrolimus (a calcineurin inhibitor) on the AMPK-MCOLN1-calcineurin signaling pathway and limb survival. Our key results demonstrated that FGF21 facilitated the AMPK-MCOLN1-calcineurin signal and enhanced the nuclear translocation of TFEB. Intriguingly, those promising efficacy were reversed by CC and tacrolimus (Fig. 1r, t and Supplementary Fig. S11d, i). CC remarkably obstructed FGF21-mediated reinforcement of autophagic activity, OS repression and subsequent inhibition of apoptosis in skeletal muscle after I/R (Fig. 1s and Supplementary Fig. S11e). Furthermore, CC and tacrolimus reversed the protective effect of FGF21 on limb perfusion and muscle edema after I/R injury, demonstrated that activation of the AMPK-MCOLN1-calcineurin signaling pathway enhances limb survival (Supplementary Fig. S11f–h, j–l). Collectively, our results verify that FGF21 enhanced TFEB activity in skeletal muscle after I/R through the AMPK-MCOLN1-calcineurin signaling pathway.

To sum up, depletion of FGF21 exacerbates microvascular damage and skeletal muscle fiber injury after I/R injury in limbs. In contrast, replenishment of FGF21 could reverse the adverse effects. Mechanistically, FGF21 enhances the nuclear translocation of TFEB via activating the AMPK-MCONL1-calcineurin signaling pathway. More importantly, FGF21-induced activation of TFEB restores the autophagic flux, especially lysosomal function and enhances the NFE2L2-mediated suppression of OS in ECs of I/R limbs. Those events induced following suppression of apoptosis in ECs, which decreases the I/R-induced microvascular damage and facilitates limb viability (Fig. 1u). Collectively, these results imply that FGF21 is a prospective therapeutic target for treatment aiming at microvascular in limb I/R injury.

## Supplementary information

Supplementary Materials

## Data Availability

Data are available upon reasonable request.
