# Supplementary material for: FGF21 alleviates microvascular damage following limb ischemia/reperfusion injury by TFEB-mediated autophagy enhancement and anti-oxidative response
Source: Signal Transduct Target Ther. 2022 Oct 12;7:349. doi: 10.1038/s41392-022-01172-y (PMC9553877; doi:10.1038/s41392-022-01172-y)
Supplement: Supplementary file 1 — Supplementary Materials [file 41392_2022_1172_MOESM1_ESM.docx]

Supplementary Materials for

**FGF21 alleviates microvascular damage following limb ischemia/reperfusion injury by TFEB-mediated autophagy enhancement and anti-oxidative response**

Fanfeng Chen^1,2#^, Jiayu Zhan^2#^, Xiaoqing Yan^2#^, Abdullah Al Mamun^2^, Yu Zhang^2^, Yitie Xu^2^, Hongyu Zhang^2^, Xiaokun Li^2*^, Kailiang Zhou^3*^, Jian Xiao ^1,2*^

^1^ Oujiang Laboratory (Zhejiang Lab for Regenerative Medicine, Vision and Brain Health), Department of Vascular Surgery, The First Affiliated Hospital of Wenzhou Medical University, Wenzhou 325015, China;

^2^ Molecular Pharmacology Research Center, School of Pharmaceutical Science, Wenzhou Medical University, Wenzhou 325000, China;

^3^ Department of Orthopaedics, The Second Affiliated Hospital and Yuying Children's Hospital of Wenzhou Medical University, Wenzhou 325027, China

^#^ Fanfeng Chen, Jiayu Zhan and Xiaoqing Yan contribut equally.

^*^ Correspondence:

^*^Jian Xiao, E-mail: xfxj2000@126.com,

^*^Kailiang Zhou, E-mail: zhoukailiang@wmu.edu.cn,

^*^Xiaokun Li, E-mail: xiaokunli@wmu.edu.cn

**This file includes:**

Materials and Methods

References

Figures. S1 to S11

**MATERIALS AND METHODS**

**Animals**

The entire experimental methods and procedures followed the Animal Care and Use Committees guidelines approved by the Animal Research Ethics Committee of Wenzhou Medical University (wydw 2017–096). Male FGF21-KO mice with C57BL/6 background were gifted from Dr Steve Kliewer, University of Texas Southwestern Medical Center. Male WT C57BL/6 mice (average bodyweight 25~30g) were mainly received from the Experimental Animal Center (no. SCXK 2005–0019) of Wenzhou Medical University, Zhejiang Province, China. All animals were placed under controlled, identical specific pathogen-free standard environmental conditions (23 ± 2 °C, 12-hour light/12-hour dark cycle) with freely access to water and foods.

**RNA interference**

TFEB was downregulated by shRNA or siRNA techniques. For our animal experiments, the adeno-associated virus (AAV)-TFEB shRNA and the control virus were formed and packaged by GeneChem Chemical Technology Co., Ltd. (Shanghai, China). AAVs were locally delivered via intramuscular injections of the hind limb gastrocnemius at 5×10^10^ vg/injection site 14 days prior to surgery. For cell experiments, siRNAs were developed and synthesized by GenePharma Company (Shanghai, China). According to the manufacturer's instructions, siRNAs were transfected into HUVECs using liposome transfection reagent 3000 (Thermo Fisher). Posterior to the 48 h transfection, the expression of TFEB was identified via qPCR.

**Groups, and treatments**

FGF21-KO mice were allocated into the sham group, I/R group and I/R+Z-DEVD-FMK group. C57BL/6 mice were distributed into sham group, I/R group, I/R+Z-DEVD-FMK, I/R+FGF21 group, I/R+FGF21/ML385 group, I/R+ML385group, I/R+FGF21/CQ group, I/R+CQ group, I/R+FGF21/TFEB shRNA group, I/R+FGF21/scrambled shRNA, I/R+FGF21/TFEB shRNA/SFN group, I/R+FGF21/SFN group, I/R+TFEB shRNA group, I/R+FGF21/CC group, I/R+CC group, I/R+FGF21/tacrolimus group and I/R+tacrolimus group. FGF21 was supplied from the laboratory of Biotechnology Pharmaceutical Engineering at Wenzhou Medical University and synthesised on the basis of the previously reported study [1]. The FGF21 group was exposed to FGF21 (1.5mg/kg, every day) via intraperitoneal injections at three time points: right before ischemia, prior to reperfusion and 30 min post-reperfusion. The I/R+FGF21/TFEB shRNA and I/R+TFEB shRNA groups received the direct intramuscular injections of an AAV-expressing TFEB shRNA in 3 sites of the right gastrocnemius muscle 14 days prior to the operation. The scramble control group was treated with the identical volume of AAV vehicle that expressing scrambled sequence as a negative control. The I/R+Z-DEVD-FMK group received with intraperitoneal injections of Z-DEVD-FMK (5 mg/kg, MCE, HY-12466) from 3 days prior to the surgery. I/R+FGF21/ML385 and I/R+ML385 groups received with intraperitoneal injections of ML385 (30 mg/kg, MCE, HY-100523) with the identical protocols. Mice from I/R+FGF21/CQ and I/R+CQ groups were treated with the intraperitoneal injections of CQ (60 mg/kg, Aladdin, C193834) with the same protocol. I/R+FGF21/TFEB shRNA/SFN group and I/R+FGF21/SFN groups received with intraperitoneal injections of Sulforaphane (SFN, 25mg/kg, Sigma Aldrich, S4441) with the identical protocols. I/R+FGF21/CC and I/R+CC groups were treated with intraperitoneal injections of CC (1.5 mg/kg, MCE, HY-13418A), and I/R+FGF21/tacrolimus and I/R+tacrolimus groups received intraperitoneal injections of tacrolimus (1 mg/kg, MCE, HY-13756) with the same protocol.

As previously explained, hind-limb ischemia was induced by using a 6.0-oz orthodon-tic elastic band around the upper thighs [2]. In brief, experimental mice were anesthetized by 0.5% sodium pentobarbital liquor (0.02 ml/g, ip). After 4 h of ischemia, the bands were eliminated and the mice were permitted to recover from anesthesia. Mice were afterwards observed for 24 h post-procedure (reperfusion interval). At the end of the reperfusion interval, mice underwent laser doppler imaging (LDI) during general anesthesia. The sham group were only received anesthesia and laser doppler pictures were acquired from every mouse 30 min posterior to induction of anesthesia. Posterior to LDI, mice were euthanised and hind-limb muscular tissue samples were stored for the investigation later.

**Cell culture and treatments**

HUVECs were [achieve](https://synonyms.reverso.net/synonym/en/achieve)d from the Molecular Pharmacology Research Center, School of Pharmaceutical Science, Wenzhou Medical University, Zhejiang Province, China. HUVECs were cautiously cultivated in Dulbecco's Modified Eagle's Medium added with 10% FBS (fetal bovine serum), 100 U/mL penicillin and 100 μg/mL kyowamycin under 37 °C in a humidified cell incubating device with continuous 5% CO_2_ supply.

H/R model was constructed when the cells were in the log-growth stage. The cultivation intermediary was displaced by a hypoxic intermediary in the absence of FBS and glucose (GLU) and the cultivation plate was put into a sterilised hypoxic box. Oxygen concentration was sustained at 1% with a corrected gaseous mix of 95% nitrogen and 5% CO_2_. Next, the box was cultivated under 37 °C in an incubating device for 8 h. Cells were cultivated in standard medium for 16 h with 4.5 g/ml GLU under 37 °C for re-oxygenation. Before H/R, cells were exposed to FGF21 (100 nM) for 24 h. Cells were subjected to TFEB siRNA transfection for 48 h or induced with ML385 (5 μM) for 24 h with or without FGF21.

As per the supplier's instructions, cell viability was identified via a CCK-8 assay (C0042, Beyotime, Shanghai, China). The value of OD450 normalized to that of the control group was used to reflect cell viability.

**LDI**

Under standardized conditions, blood perfusion in the limb was monitored by LDI [3]. At 24 h post-reperfusion, mice were transferred into a reliable and standard environment after anesthesia. Afterwards, the blood flow of the limbs was identified via a laser doppler apparatus (Moor Instruments, United Kingdom). Blood flow was studied on the foundation of perfusion units (PU) computed via Moor LDI Review program 6.1. All mice were identified in triplicate and the mean value was adopted for the subsequent statistic assay.

**Determination of tissue edema**

Right after the collection, tissue specimens were subjected to blotting, weighing, and put in desiccation stove under 55 °C till an unchanged was achieved. Skeletal muscular oedema was identified via measuring the wet to dry weight ratio.

**Quantitative assessment of skeletal muscle fiber injury**

Gastrocnemius muscle tissues were collected after 24 h of reperfusion. Posterior to the fixation with 4% (w/v) paraformaldehyde (PFA) for 24 h, samples were sufficiently dehydrated in graded ethanol solution, subjected to paraffin embodiment and sliced into 2 μm slices. The tissue slices were adequately stained with Masson's trichrome. A blinded observer analyzed skeletal muscle fiber injury performing the full-frame counting method as previously established [4]. Muscular fibers were counted and scored as non-injured/injured on the foundation of the morphology of the separate fiber as previously described [4]. Summarized data were presented as the percentage of injured fibers in every limb.

**Immune fluorescence dyeing of HUVECs and gastrocnemius muscle slices**

HUVECs were cultivated on gelatinised coverslips for one night. Posterior to the aforesaid experiment process, cells were subjected to fixation with 4% PFA in phosphate-buffered saline (PBS) for 15 min, and punched with 0.5% Triton for 20 min. Subsequently, the cells were cultivated with specific primary antibodies against NFE2L2 (1:200, proteintech, 16396-1-AP) and TFEB (1:200, Thermo Fisher, PA5-96632) at 4 °C for overnight. To evaluate LAMP1 and CTSD localisation, cells were subjected to the treatment as aforementioned and afterwards dyed with CTSD (1:200, ZEN Bio, 380946) and LAMP1 (1:200, Thermo Fisher, 12-1071-81) overnight. Then, the dyed slides were inoculated with Alexa Flour 488-conjugated anti-rabbit secondary antibody (Abcam, ab150073) and Alexa fluor 647-conjugated anti-mouse secondary antibody (Abcam, ab150107) at a dilution of 1: 400 for 1 h under ambient temperature. Cellular nuclei were afterwards subjected to DAPI reagent labeling for 60 min.

For gastrocnemius muscle staining, sections were cultivated for one night under 4 °C with primary antibodies against CD31 (1:200, Servicebio, GB11063-2), α-SMA (1:200, Abcam, ab7817), HO1 (1:200, proteintech, 10701-1-AP), NFE2L2 (1:200, proteintech, 16396-1-AP), CTSD (1:200, ZEN Bio, 380946), p62 (1:200, proteintech, 18420-1-AP) and TFEB (1:200, Thermo Fisher, PA5-96632). After sufficient washing with PBS solution, specimens were cultivated with Alexa fluor 647-conjugated anti-mouse IgG secondary antibody and/or Alexa fluor 488-conjugated anti-rabbit IgG secondary antibody at a dilution of 1: 400 for 60 min under ambient temperature. Cell nuclei subjected to DAPI labeling. Eventually, digitalized pictures were obtained via the Nikon ECLIPSE 80i microscopic device (Japan).

**Measurement of ROS level**

In vivo, ROS production was evaluated by DHE staining. Briefly, cryosections of fresh skeletal muscle samples were stained with DHE (5 μmol/L, Sigma Aldrich, D7008) in the dark at 37 °C for 30 min, and then were visualized in a blinded manner under a confocal microscope (Nikon, Japan).

In vitro, ROS levels were quantified by applying the ROS Assay Kit (Beyotime, S0033, China) as per the supplier's specification. HUVECs were isolated and suspended in desaturated DCFH-DA, an oxidation-susceptible fluorescence probe and cultivated under 37 °C for 20 min in 5% CO_2_. Posterior to the [adequate](https://synonyms.reverso.net/synonym/en/adequate) washing with a serum-free medium, cellular fluorescent results were instantly identified by a confocal microscope (Nikon, Japan).

**TUNEL assay**

HUVECs were carefully subjected to fixation in PBS with 4% PFA. Gastrocnemius muscle from every mouse was attentively subjected to fixation in PBS with 4% PFA, afterwards treated with paraffin embedment. The paraffinised samples were sliced (5 µm) and embedded on slides. Staining was performed using the TUNEL ApoGreen Detection Kit (Yeasen, 40307ES50) as per the supplier's specification.

**Transcriptome sequencing**

Total RNA was abstracted and a genome-wide transcriptomics analyses were finished (LC-BIOTECHNOLOGIS (HANGZHOU) CO., LTD). The differently expressed mRNAs were screened with fold change (FC) > 2 or FC < 0.5 and p-value < 0.05 by R package edgeR (https://bioconductor.org/packages/release/bioc/html/edgeR.html) or DESeq2 (http://www.bioconductor.org/packages/release/bioc/html/DESeq2.html). Hierarchical cluster analyses of DEGs were completed to identify the expressing patterns of the genes in diverse groups. GO enrichment analyses of DEGs were separately completed via R on the foundation of the hypergeometrical distribution. Complete data were uploaded to NCBI database (BioProject ID: PRJNA797180).

**Real-time quantitative PCR**

Total RNA from muscle tissues were abstracted by Trizol reagent (Sangon Biotech) as per the supplier's specification. The cDNA was constructed via TransScript One-Step gDNA Removal and cDNA synthesis SuperMix (TransGen) and then applied as templates for reverse transcription. Real-time PCR was completed via specific primers by the nexus GSX1 Mastercycler (Eppendorf). The reactive volume was set to 20 μl and completed under 50 °C for 120 s, 95 °C for 20 s, followed by 40 cycles of 3 s under 95 °C and 30 s under 60 °C. Data studied via the 2-ΔΔCt method. The mRNA levels of target genes were normalized to β-actin levels. Gene-specific primer sequences utilized for real-time-PCR were synthesized by GENERAY Biotechnology and listed below: *Ho1, 5’- GATAGAGCGCAACAAGCAGAA-3’ (For), and 5’-CAGTGAGGCCCATACCAGAAG-3’ (Rev); NQO1, 5’- AGGATGGGAGGTACTCGAATC-3’ (For), and 5’-TGCTAGAGATGACTCGGAAGG-3’ (Rev); Sod1, 5’- AACCAGTTGTGTTGTCAGGAC-3’ (For), and 5’-CCACCATGTTTCTTAGAGTGAGG-3’ (Rev); Gpx3, 5’- CCTTTTAAGCAGTATGCAGGCA-3’ (For), and 5’-CAAGCCAAATGGCCCAAGT-3’ (Rev); Nfe2l2, 5’- TAGATGACCATGAGTCGCTTGC-3’ (For), and 5’-GCCAAACTTGCTCCATGTCC-3’ (Rev); Lamp1, 5’- GACGGTGACCAGAGCGTTC-3’ (For), and 5’-GTGGGCACTAGGGCATCAG-3’ (reverse); Beclin1, 5’- ATGGAGGGGTCTAAGGCGTC-3’ (For), and 5’-TGGGCTGTGGTAAGTAATGGA-3’ (Rev); p62, 5’- GAACTCGCTATAAGTGCAGTGT-3’ (For), and 5’-AGAGAAGCTATCAGAGAGGTGG-3’ (Rev); Ctsd, 5’- GCTTCCGGTCTTTGACAACCT-3’ (For), and 5’-CACCAAGCATTAGTTCTCCTCC-3’ (Rev); Atg5, 5’- TGTGCTTCGAGATGTGTGGTT-3’ (For), and 5’-ACCAACGTCAAATAGCTGACTC-3’ (Rev); Lc3II, 5’- TTATAGAGCGATACAAGGGGGAG-3’ (For), and 5’-CGCCGTCTGATTATCTTGATGAG-3’ (**Rev);* *β-actin, 5’- GGCTGTATTCCCCTCCATCG-3’ (For), and 5’-CCAGTTGGTAACAATGCCATGT-3’ (Rev).*

**WB analyses**

Skeletal muscle tissue and cell samples were dissected and treated via abstracting proteins via a lysis buffering reagent. Subsequently, the protein concentration was identified via a BCA protein assay kit (Thermo Fisher Scientific). The protein specimens were isolated on 12% sodium dodecyl sulfate-polyacrylamide gel electrophoresis and placed onto a polyvinylidene fluoride membranes (Millipore). Later, the membranes were subjected to blockade in 10% non-fat milk, the membranes cultivated with the antibodies below overnight under 4 °C: FGF21 (1:1,000, Abclonal, A3908), HO1 (1:1,000, proteintech, 10701-1-AP), NQO1 (1:1,000, proteintech, 11451-1-AP), SOD1 (1:1,000, proteintech, 10269-1-AP), NFE2L2 (1:1,000, proteintech, 16396-1-AP), LAMP1 (1:1,000, Santa, SC-20011), BECLIN1 (1:1,000, proteintech, 11306-1-AP), CTSD (1:1,000, ZEN Bio, 380946), p62 (1:500, proteintech, 18420-1-AP), LC3 (1:1,000, proteintech, 14600-1-AP), TFEB (1:1,000, Thermo Fisher, PA5-96632), Bax (1:1,000, ZEN Bio, R22708), Bcl-2 (1:1,000, ZEN Bio, 383309), CC3 (1:1,000, proteintech, 19677-1-AP), AMPKα (1:1,000, ZEN Bio, 380431), AMPKα (1:1,000, ZEN Bio, 381164), MCOLN1 (1:1,000, Invitrogen, PA1-46474), Calcineurin (1:1,000, ZEN Bio, R26497), GAPDH (1:1,000, proteintech, 10494-1-AP), Histone H3 (1:1,000, ZEN Bio, 384572). Subsequently, the membrane was cultivated with HRP-conjugated IgG second antibody under ambient temperature for 2 h. The bands were finally visualized and evaluated via Image Lab 3.0 program (Bio-Rad)

**Luciferase assay**

A luciferase reporter assay was performed to identify whether FGF21 regulates the transcriptional activity of TFEB. TFEB recognises a specific CLEAR motif (GTCACGTGAC) enriched in the promotor regions of some lysosome and autophagy genes such as CTSD [5]. Therefore, the transcriptional activity of TFEB was detected by the luciferase activities driven by the CTSD promotor which contains the CLEAR element. HEK293T cells were carefully co-transfected with pRL-TK, pGL3-CLEAR box WT or pGL3-CLEAR box MUT and empty pcDNA3.1 vector or TFEB-pcDNA3.1 plasmids in 24-well plates with liposome transfection reagent 2000 (Invitrogen, Carlsbad, CA) as per the supplier's specification. Posterior to the 24 h post-transfection, the cells were co-treated with FGF21 (40 nM or 100 nM). After 48 h post-transfection, firefly luciferase activity and Renilla luciferase activities were analyzed by performing the Dual-Luciferase® Reporter Assay System (Promega) with a microplate reading device (Synergy H1, Bio-Tek) and the ratio of firefly/Renilla luciferase was measured.

**Statistical analyses**

For all quantified data, mean ± standard deviation (SD) is presented. Statistical difference was evaluated via the non-paired 2-tailed Student's t-test for 2 experimental groups and one-way ANOVA for several groups via SPSS program version-19 (Chicago, IL, USA). Bonferroni's post-hoc testing was completed posterior to ANOVA for the significant [deviation](https://synonyms.reverso.net/synonym/en/deviation) between groups. A two-tailed *p*-value of less than 0.05 had significance on statistics.

**REFERENCES**

1. Wang H, et al. High-level expression and purification of soluble recombinant FGF21 protein by SUMO fusion in Escherichia coli. BMC Biotechnol. **10**, 14 (2010).

2. Crawford RS, et al. A novel model of acute murine hindlimb ischemia. Am J Physiol Heart Circ Physiol. **292**, 830-837 (2007).

3. Dick F, et al. Basic control of reperfusion effectively protects against reperfusion injury in a realistic rodent model of acute limb ischemia. Circulation. **118**, 1920-1928 (2008).

4. McCormack MC, et al. Development of reproducible histologic injury severity scores: skeletal muscle reperfusion injury. Surgery. **143**, 126-133 (2008).

5. Palmieri M, et al. Characterization of the CLEAR network reveals an integrated control of cellular clearance pathways. Hum Mol Genet. **20**, 3852-3866 (2011).

**Supplemental Figures**

**Figure S1**

**
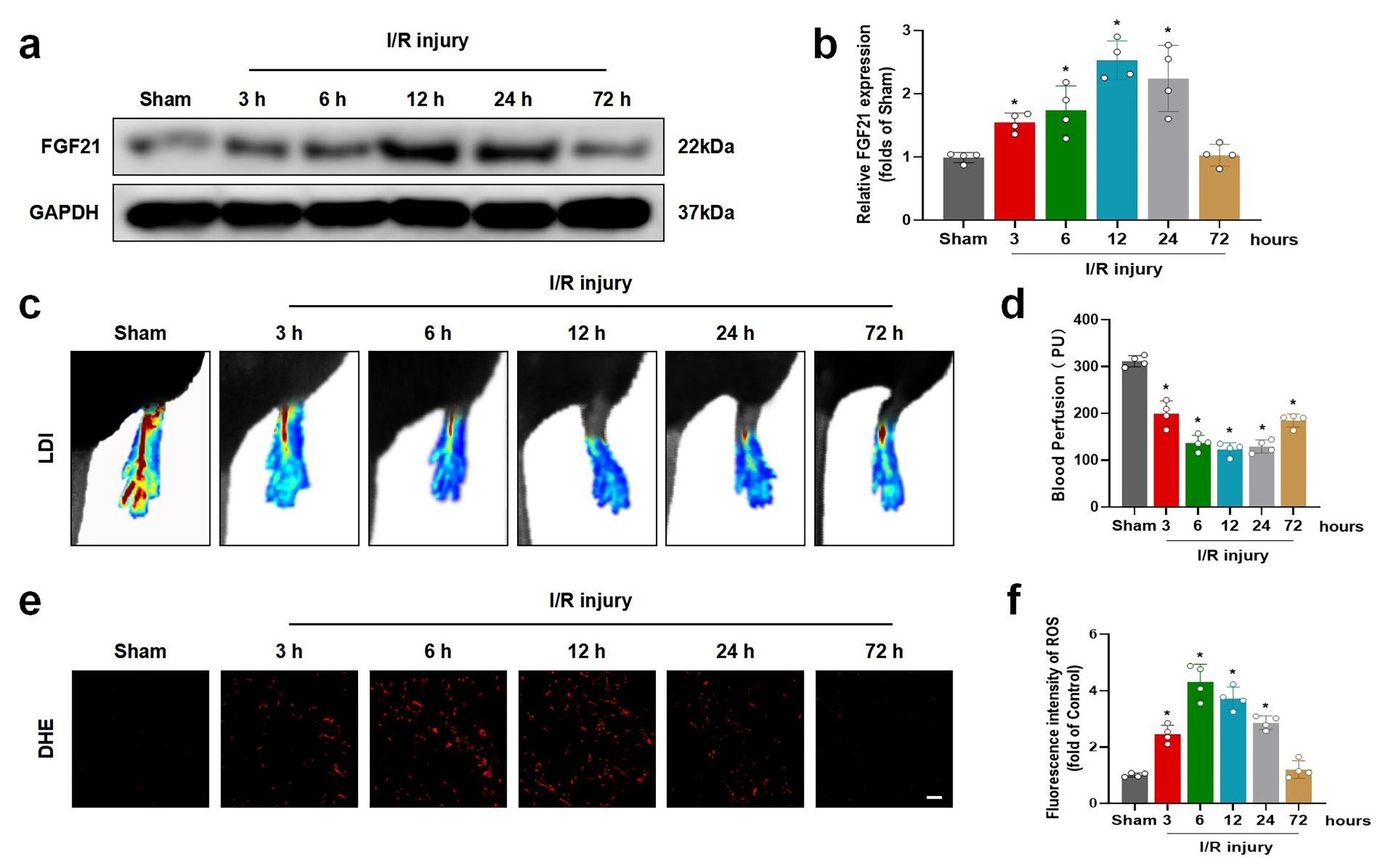
**

**Figure S1.** Changes of FGF21 level, blood perfusion and ROS level after I/R injury in limbs. (**a**) Western blot showing FGF21 levels at several time points after I/R injury. (**b**) Densitometric analysis of FGF21 from (a) with normalized to the loading control GAPDH. (**c**) Blood perfusion of FGF21-KO and WT mice before and after I/R injury were detected by LDI. (**d**) Histogram showing signal intensity of blood ﬂow in I/R limbs. (**e**) ROS level in skeletal muscle was detected by DHE staining assay; scale bars: 100 μm. (**f**) Quantification of immunofluorescence data from (e) showing the mean optical density of ROS. Data are expressed as the means ± SD (n = 4 per group). Significance: ^*^*P* < 0.05.

**Figure S2**


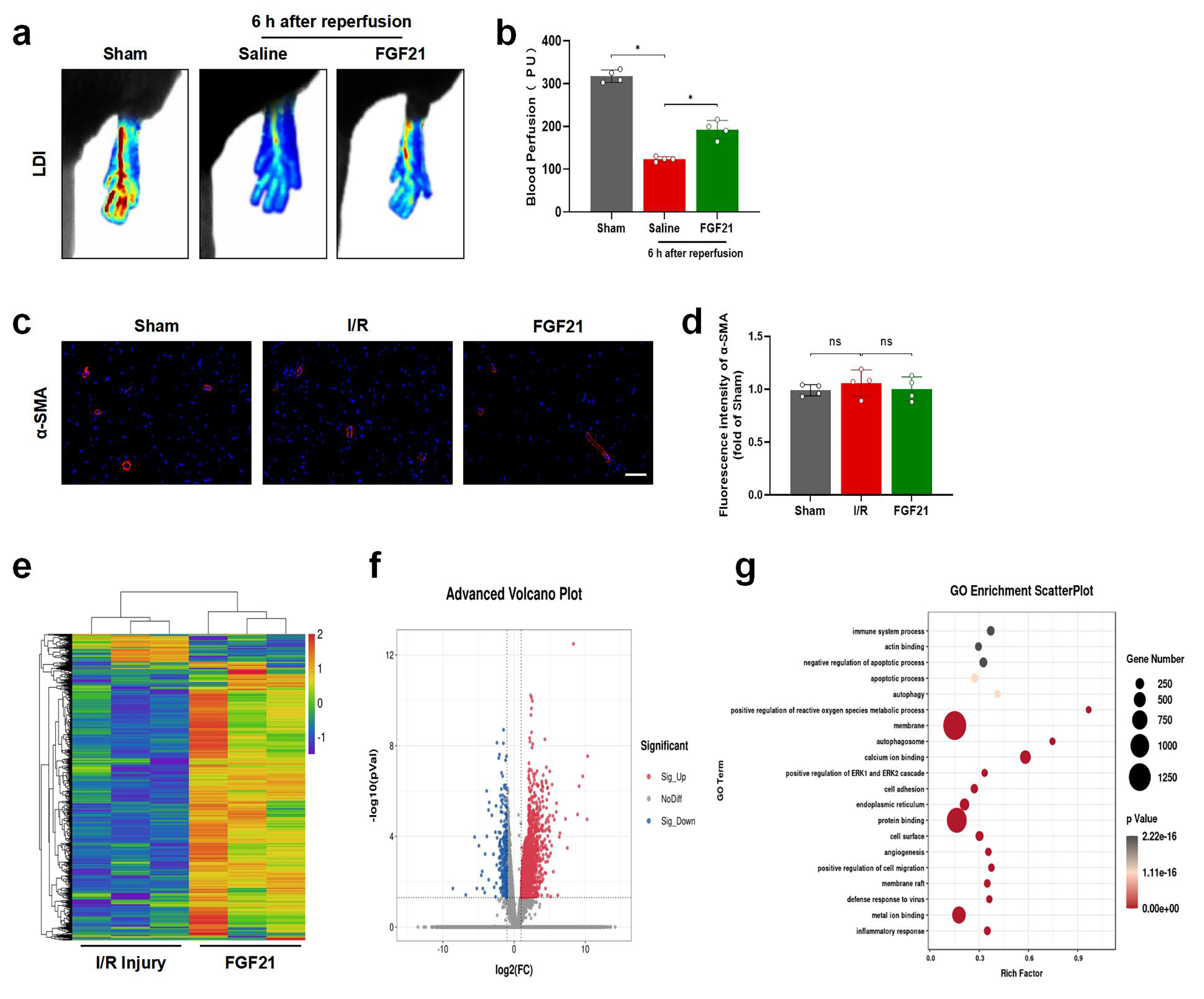


**Figure S2.** FGF21 enhances blood perfusion and microvascular density in I/R limbs.

(**a**) Blood perfusion of hind limbs were detected by LDI. (**b**) Signal intensity of blood ﬂow was plotted as a histogram. (**c**) Images of skeletal muscle sections stained with antibodies against α-SMA; scale bar: 100 µm. (**d**) Quantification of immunofluorescence data from (c) displaying the average optical density of α-SMA. (**e**) Heatmap displays the distinct abundance features of I/R and I/R+FGF21 groups. (**f**) Volcano plot representing the significant variables in the discrimination between I/R and I/R+FGF21 groups. (**g**) Functional categorization of genes regulated by FGF21 using the GO database. Data are displayed as the means ± SD (n = 3-4 per group). Significance: ^*^*P* < 0.05.

**Figure S3**

**
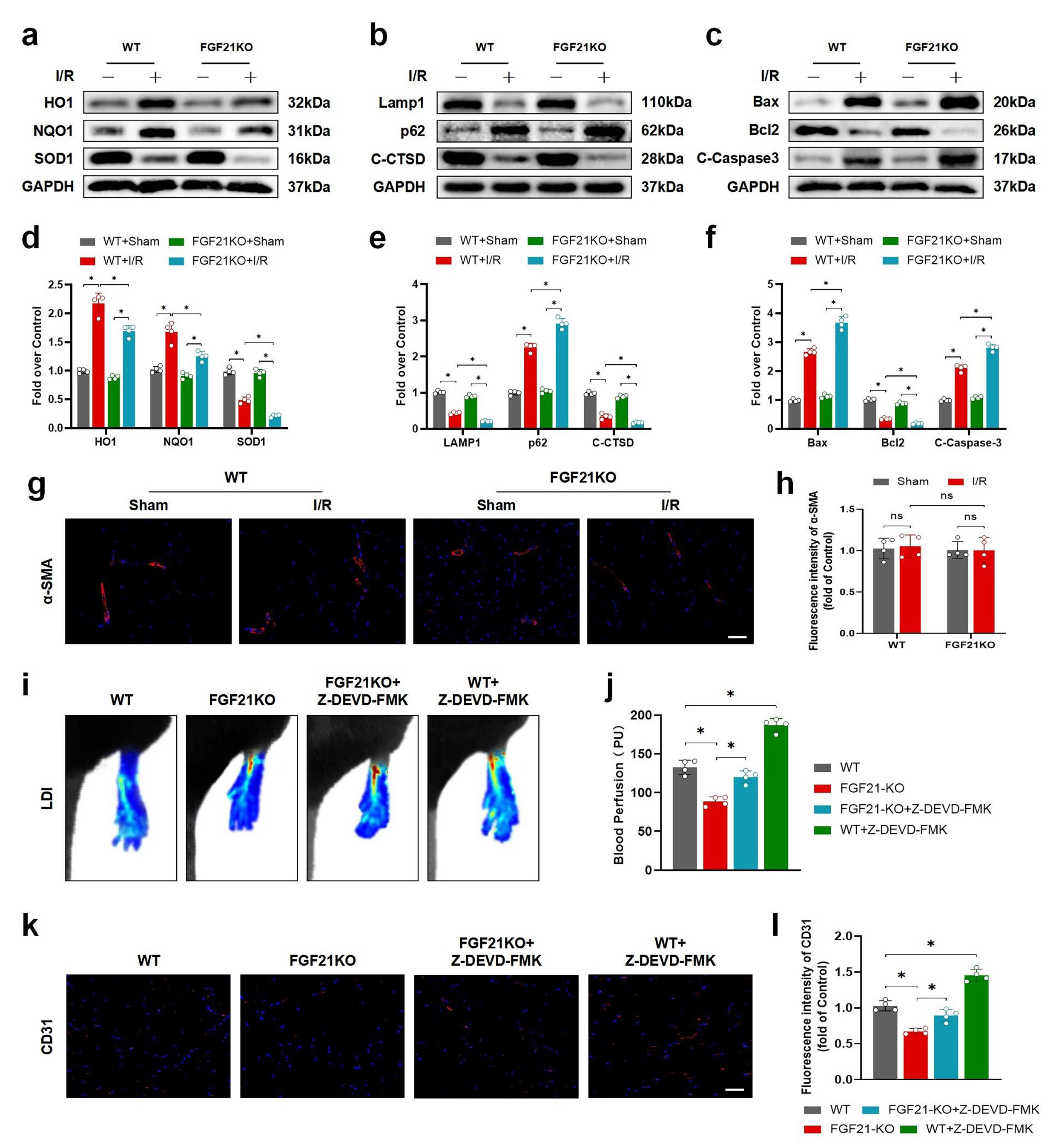
**

**Figure S3.** Depletion of FGF21 exacerbates oxidative stress, impairment in autophagy flux, and apoptosis in I/R limbs. After 24 h reperfusion, FGF21-KO mice and WT littermates were euthanized and skeletal muscle samples were harvested for the evaluation. (**a-c**) Western blots for oxidative stress (a), autophagy flux (b) and apoptosis (c) markers in skeletal muscle tissues. (**d**) Corresponding densitometric analysis of the bands from (a) normalized to the loading control GAPDH. (**e**) Corresponding densitometric analysis of the bands from (b) normalized to the loading control GAPDH. (**f**) Corresponding densitometric analysis of the bands from (c) normalized to the loading control GAPDH. (**g**) Images of skeletal muscle sections stained with antibodies against α-SMA; scale bar: 100 µm. (**h**) Quantification of immunofluorescence data from (g) displaying the average optical density of α-SMA. (**i**) Blood perfusion of hind limbs were detected by LDI in WT and FGF21 KO mice after I/R injury treated with/without Z-DEVD-FMK (a specific caspase-3 inhibitor). (**j**) Signal intensity of blood ﬂow was plotted as a histogram. (**k**) Images of skeletal muscle sections stained with antibodies against CD31; scale bar: 100 µm. (**l**) Quantification of immunofluorescence data from (k) displaying the average optical density of CD31. Data are expressed as the means ± SD (n = 4 per group). Significance: ^*^*P* < 0.05.

**Figure S4**

**
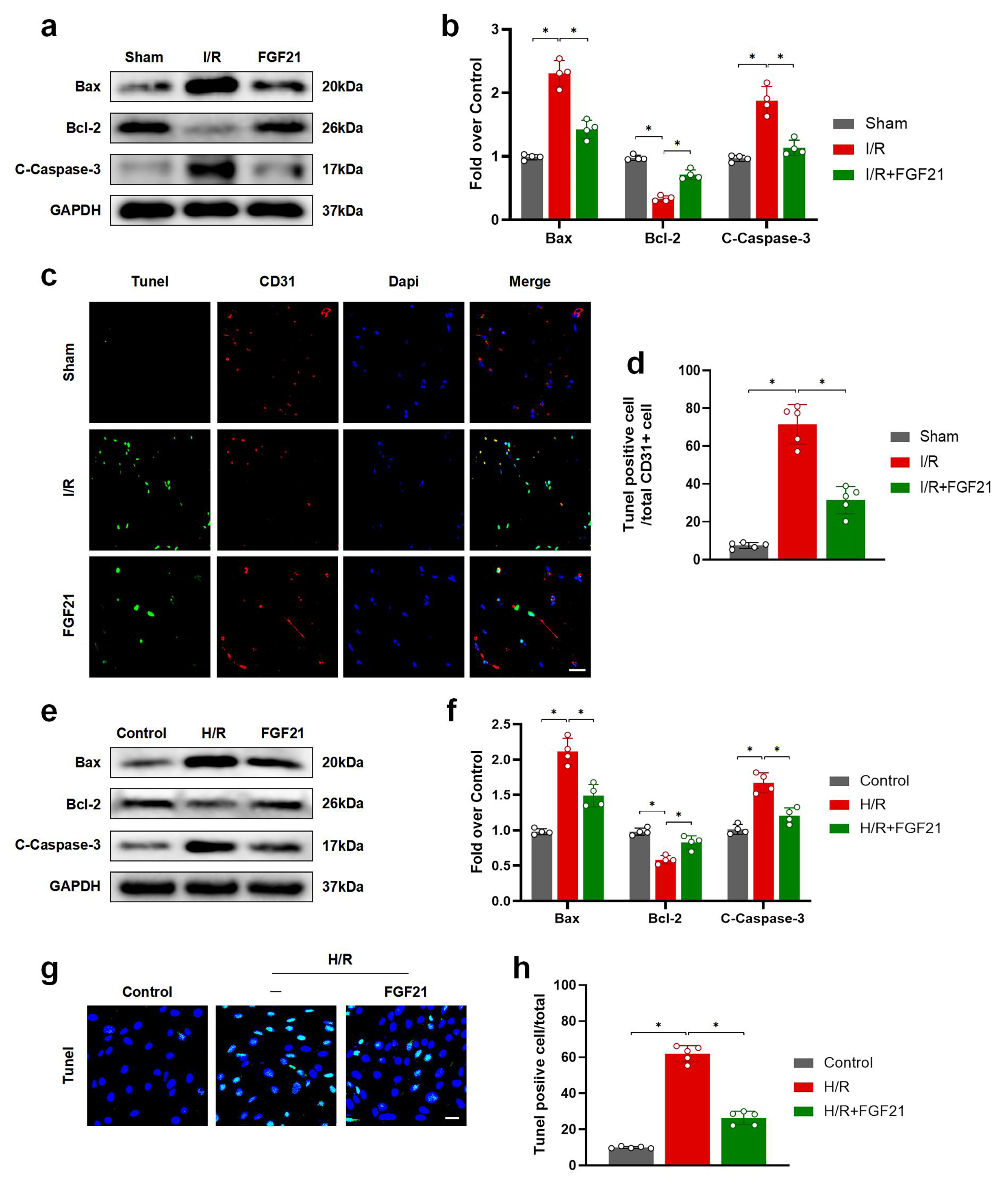
**

**Figure S4.** FGF21 suppresses I/R induced apoptosis changes in ECs. HUVECs were treated with PBS or FGF21 then underwent HR. Mice were administered with saline or FGF21 and subsequently underwent I/R. (**a**) Western blots for Bax, Bcl-2 and cleaved caspase 3 in skeletal muscle tissues. (**b**) Quantifcations of protein level of Bax, Bcl-2 and cleaved caspase 3 from (a) with normalized to the loading control GAPDH. (**c**) Images of skeletal muscle sections stained with TUNEL and EC marker CD31, merged images include DAPI staining. Scale bars: 100 μm. (**d**) Quantifcation of TUNEL and CD31 double-positive cells, the percentages of double positive cells versus total CD31 positive cells are indicated. (**e**) Western blots for Bax, Bcl-2 and cleaved caspase 3 in HUVECs. (**f**) Quantifcations of protein level of Bax, Bcl-2 and cleaved caspase 3 from (e) with normalized to the loading control GAPDH. (**g**) TUNEL assay of HUVECs, Scale bars: 25 μm. (**h**) The percentages of TUNEL positive cells versus total cells are indicated from (g). Data are expressed as the means ± SD (n = 4-5 per group). Significance: ^*^*P* < 0.05.

**Figure S5**

**
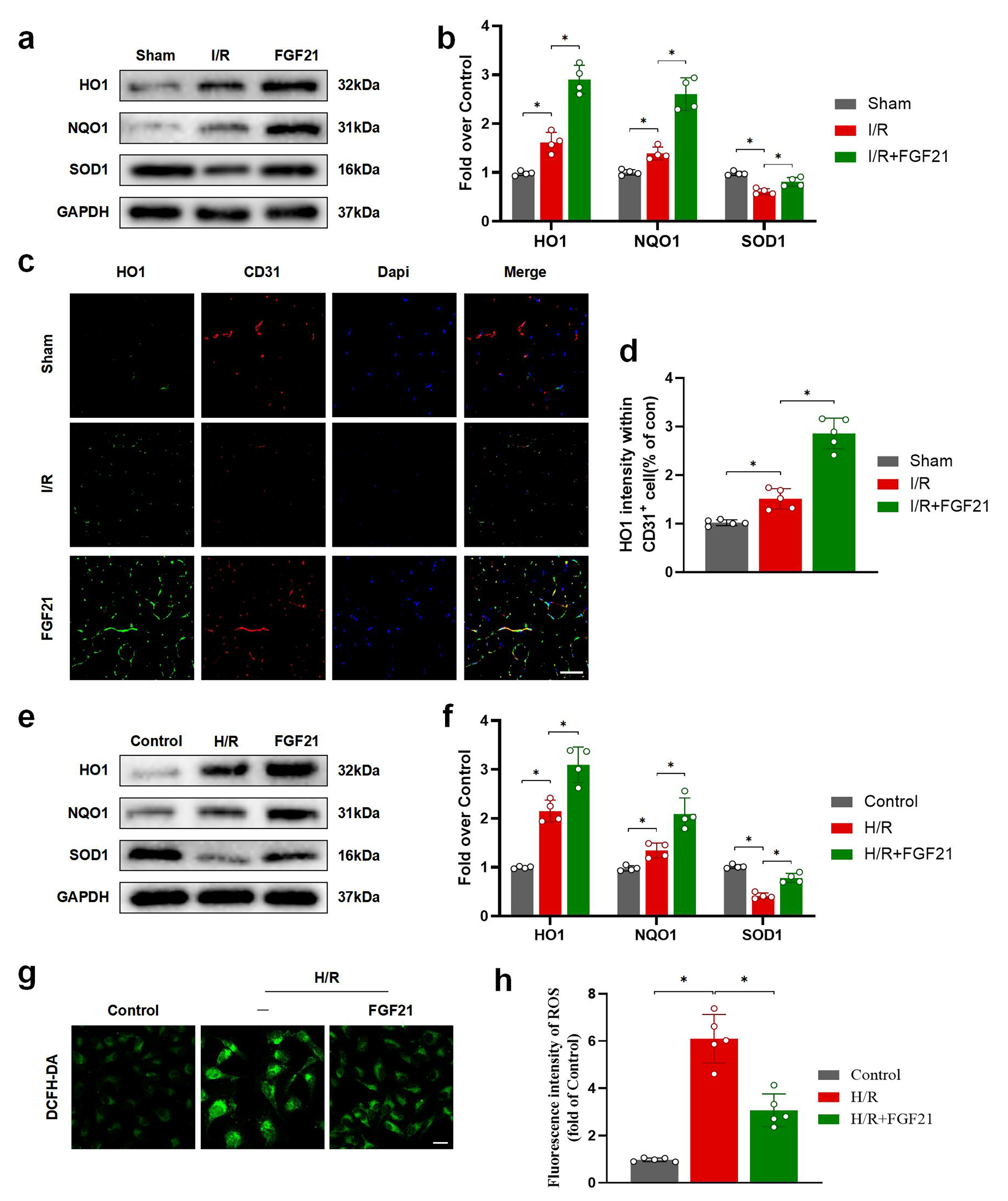
**

**Figure S5.** FGF21 attenuates I/R induced oxidative stress in ECs. HUVEC were treated with PBS or FGF21 then underwent HR. Mice were administered with saline or FGF21 and subsequently underwent I/R. (**a**) Western blots for HO-1, NQO1 and SOD1 in skeletal muscle tissues. (**b**) Quantifcations of protein level of HO-1, NQO1 and SOD1 from (a) with normalized to the loading control GAPDH. (**c**) Images of skeletal muscle sections stained with HO-1 and EC marker CD31, merged images include DAPI staining. Scale bars: 50 μm. (**d**) Quantifcations of HO-1 fuorescence intensity on vascular endothelium (CD31^+^ cell) in the skeletal muscle. (**e**) Western blots for HO-1, NQO1 and SOD1 in HUVEC. (**f**) Quantifcations of protein level of HO-1, NQO1 and SOD1 from (e) with normalized to the loading control GAPDH. (**g**) ROS of HUVECs was detected by DCFH-DA staining assay, Scale bars: 25 μm. (**h**) Quantification of immunofluorescence data from (g) showing the mean optical density of ROS. Data are expressed as the means ± SD (n = 4-5 per group). Significance: ^*^*P* < 0.05.

**Figure S6**

**
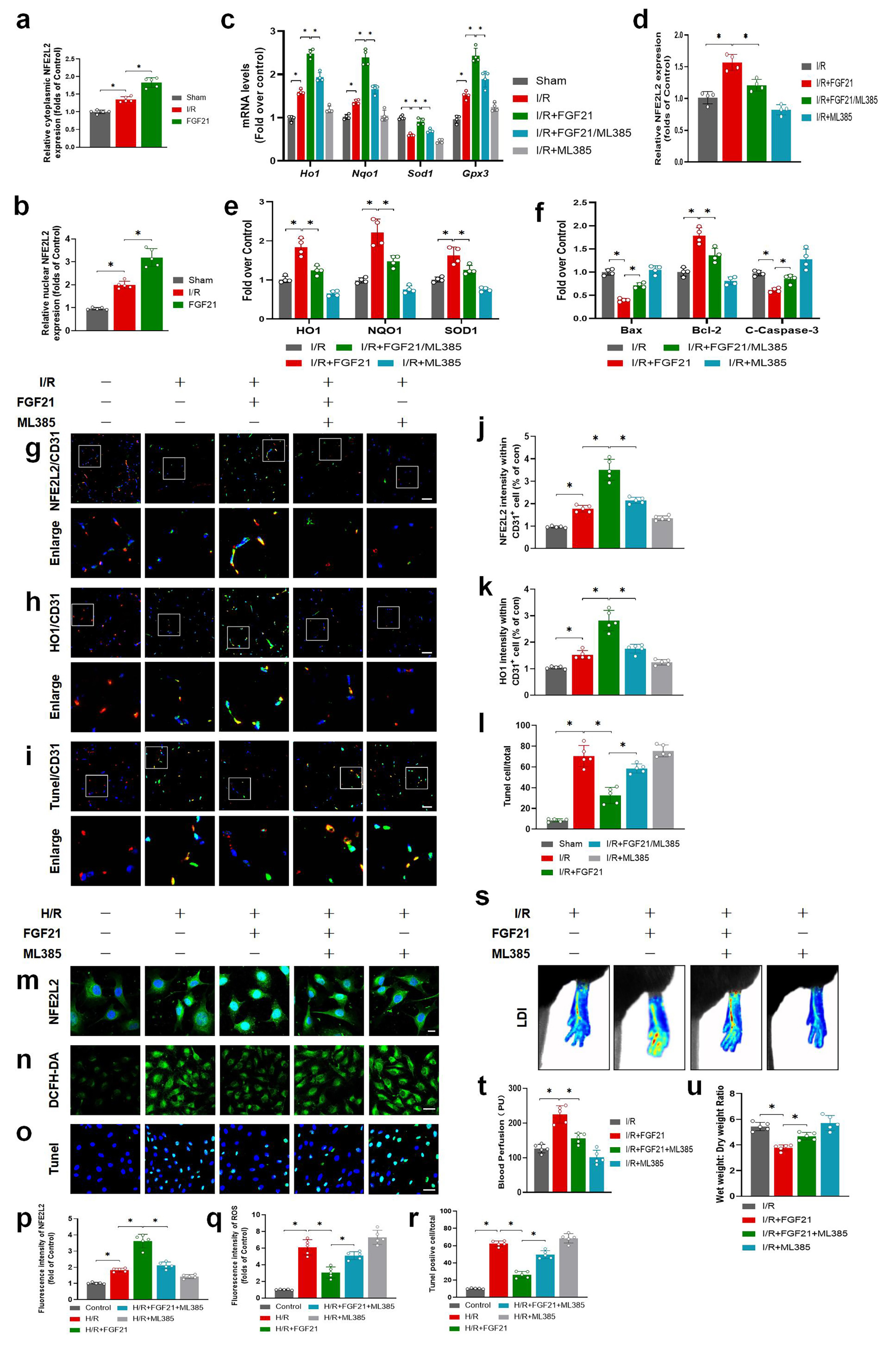
**

**Figure S6.** NFE2L2 is required for FGF21-induced removal of excessive ROS in ECs. HUVECs were treated with PBS, FGF21, FGF21+ML385 and ML385 then underwent HR. Mice were treated saline, FGF21, FGF21+ML385 and ML385 then underwent IR. (**a, b**) Quantifcations data from (Fig. 1i) showing the protein level of NFE2L2 in the cytoplasm (a) and nucleus (b). (**c**) Relative mRNA level of *Nfe2l2, Ho1, Nqo1, Sod and Gpx3* in skeletal muscle tissues of the indicated groups normalized to control β-actin. (**d**) Corresponding densitometric analysis of the bands from (Fig. 1j) normalized to the loading control Histone 3. (**e, f**) Corresponding densitometric analysis of the bands from (Fig. 1k) normalized to the loading control GAPDH. (**g-i**) Images of skeletal muscle sections stained with antibodies against NFE2L2/CD31 (g), HO-1/CD31 (h) and TUNEL/CD31 (i), merged images include DAPI staining. Scale bars: 100 μm. (**j, k**) Quantification of immunofluorescence data showing the mean optical density of NFE2L2 (g) and HO-1 (h) on vascular endothelium (CD31^+^ cell) in the skeletal muscle of mice. (**l**) Quantifcation of TUNEL and CD31 double-positive cells, the percentages of double positive cells versus total CD31 positive cells are indicated, data from (i). (**m**) Representative immunofluorescence images of HUVECs depicting NFE2L2 and DAPI. Scale bars: 25 μm. (**n**) ROS of HUVECs was detected by DCFH-DA staining assay, Scale bars: 25 μm. (**o**) TUNEL assay of HUVECs, Scale bars: 25 μm. (**p**) Quantification of immunofluorescence data from (m) showing the mean optical density of NFE2L2 in nucleus. (**q**) Quantification of immunofluorescence data from (n) showing the mean optical density of ROS. (**r**) The percentages of TUNEL positive cells versus total cells are indicated from (o). (**s**) Blood perfusion of hind limbs were detected by LDI. (**t**) Histogram showing signal intensity of blood ﬂow in I/R limbs. (**u**) Wet weight to dry weight ratio. Data are expressed as the means ± SD (n = 4-5 per group). Significance: ns stands for not significant, ^*^*P* < 0.05.

**Figure S7**

**
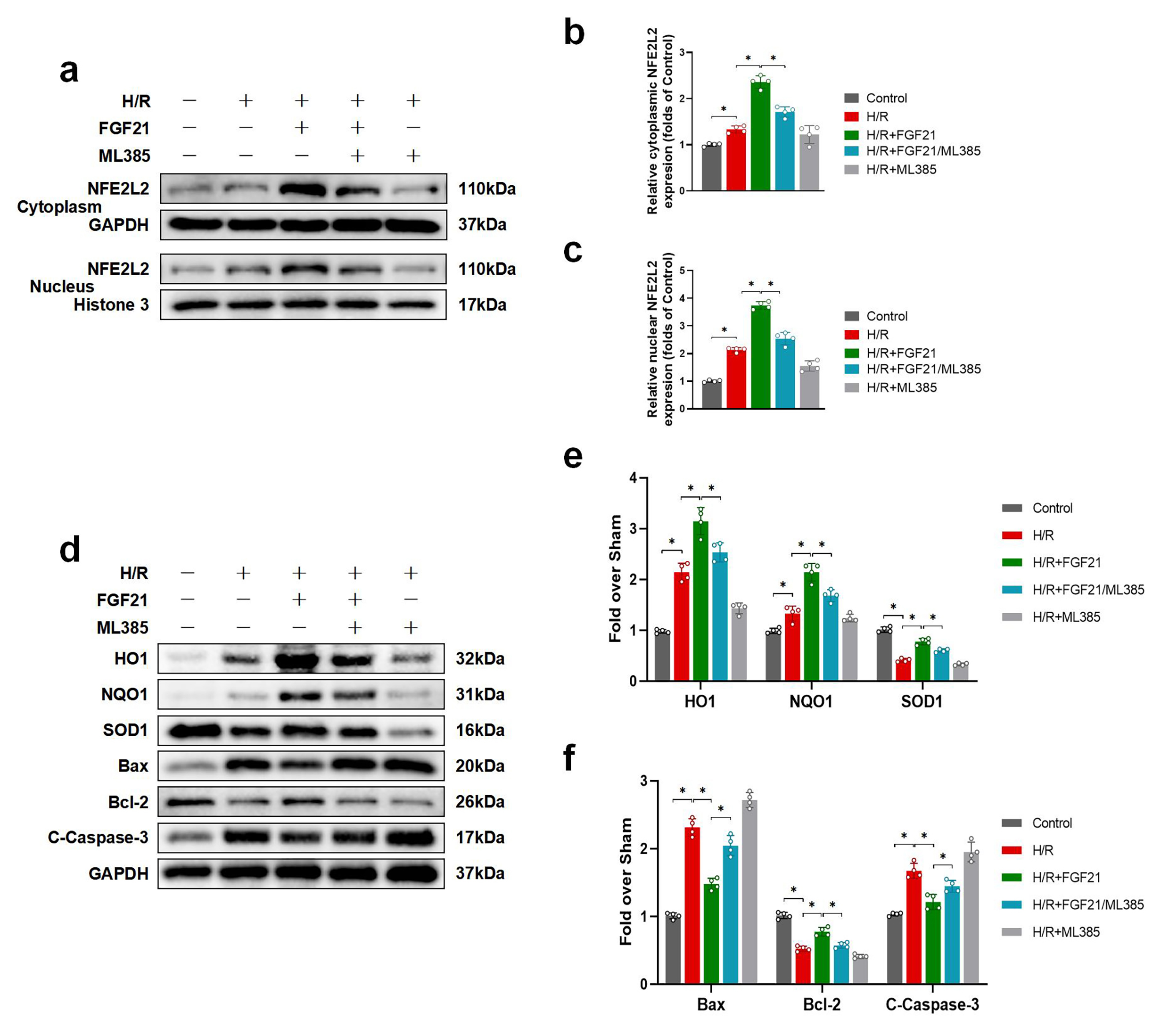
**

**Figure S7.** NFE2L2 is required for FGF21 mediated anti-oxidative and anti-apoptosis effects in vitro. HUVECs were treated with PBS, FGF21, FGF21+ML385 and ML385 then underwent HR. (**a**) Western blots for NFE2L2 in HUVECs. (**b, c**) Quantifcations data from (a) showing the protein level of NFE2L2 in the cytoplasm (b) and nucleus (c). (**d**) Western blots for oxidative stress and apoptosis markers in HUVECs. (**e, f**) Corresponding densitometric analysis of the bands from (d) normalized to the loading control GAPDH. Data are expressed as the means ± SD (n = 4 per group). Significance: ns stands for not significant, ^*^*P* < 0.05.

**Figure S8**

**
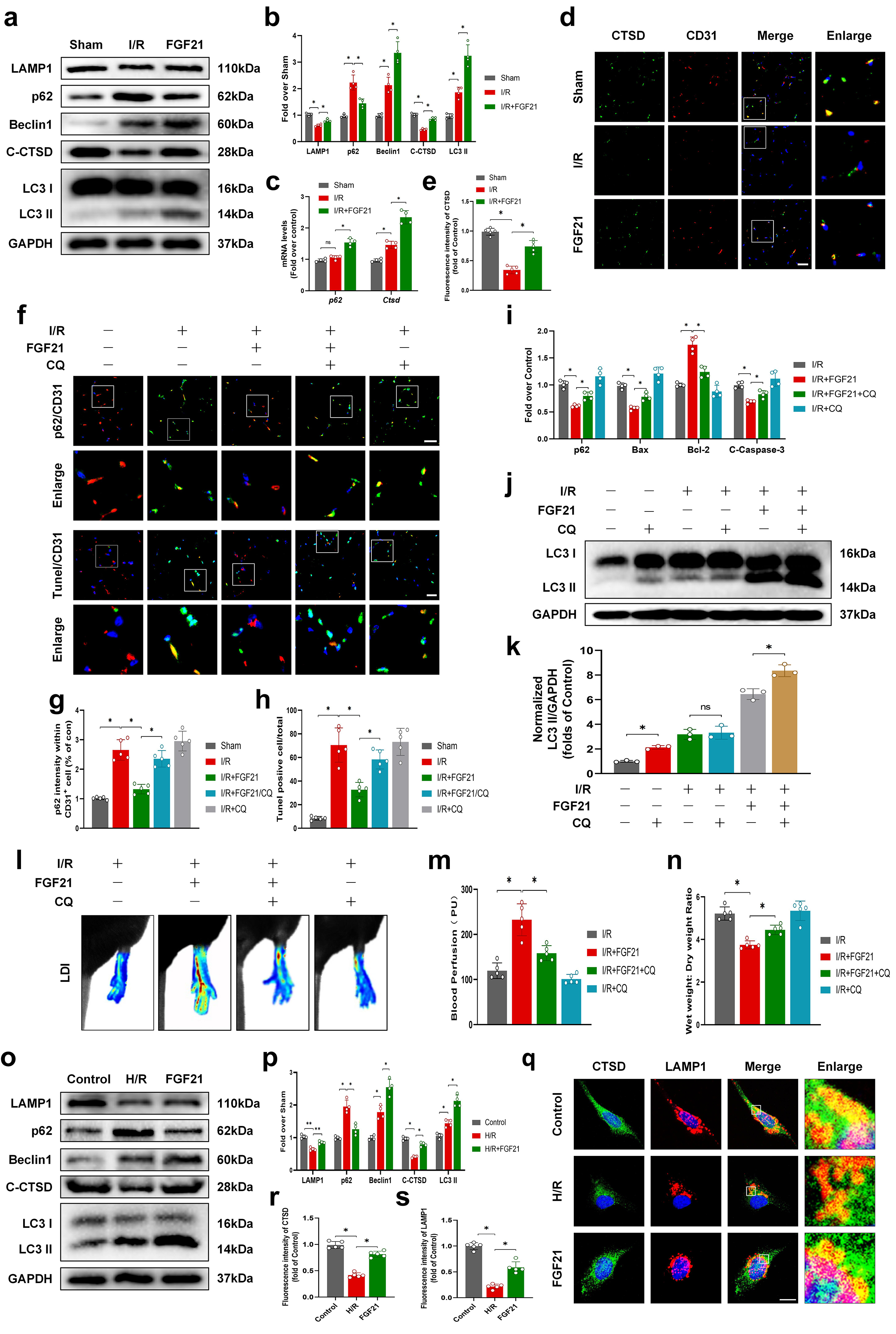
**

**Figure S8.** FGF21 restores autophagic flux associated with the amelioration of lysosomal function in ECs. HUVECs were treated with PBS and FGF21 then underwent HR. Mice were treated saline, FGF21, FGF21+CQ and CQ then underwent IR. (**a**) Western blots for autophagy flux biomarkers in skeletal muscle tissues after FGF21 administration. (**b**) Corresponding densitometric analysis of the bands from (a) normalized to the loading control GAPDH. (**c**) Relative mRNA level of *p62* and *Ctsd* in in skeletal muscle tissues of the indicated groups normalized to control β-actin. (**d**) Images of skeletal muscle sections stained with antibodies against CTSD/CD31, merged images include DAPI staining. Scale bars: 50 μm. (**e**) Quantification of immunofluorescence data from (d) showing the mean optical density of CTSD on vascular endothelium (CD31^+^ cell) in the skeletal muscle of mice. (**f**) Images of skeletal muscle sections stained with antibodies against p62/CD31 and TUNEL/CD31, merged images include DAPI staining. Scale bars: 20 μm. (**g**) Quantification of immunofluorescence data from (f) showing the mean optical density of p62 on vascular endothelium (CD31^+^ cell) in the skeletal muscle of mice. (**h**) Quantifcation of TUNEL and CD31 double-positive cells, the percentages of double positive cells versus total CD31 positive cells are indicated, data from (f). (**i**) Corresponding densitometric analysis of the bands from (Fig. 1l) normalized to the loading control GAPDH. (**j**) Western blot analysis of LC3 in Sham, I/R and I/R treated with FGF21 skeletal muscle slides at cultured in the presence or absence of CQ. (**k**) Densitometric analysis of LC3II from (j) normalized to the loading control GAPDH. (**l**) Blood perfusion of hind limbs were detected by LDI. (**m**) Histogram showing signal intensity of blood ﬂow in I/R limbs. (**n**) Wet weight to dry weight ratio. (**o**) Western blots for autophagy flux markers in HUVECs. (**p**) Corresponding densitometric analysis of the bands from (o) normalized to the loading control GAPDH. (**q**) Representative immunofluorescence images of HUVECs depicting CTSD and LAMP1, nuclei were recognized by DAPI staining. Scale bars: 10 µm. (**r-s**) Quantification of immunofluorescence intensity displaying the average optical density of CTSD and LAMP1. Data are expressed as the means ± SD (n = 3-5 per group). Significance: ns stands for not significant, ^*^*P* < 0.05.

**Figure S9**

**
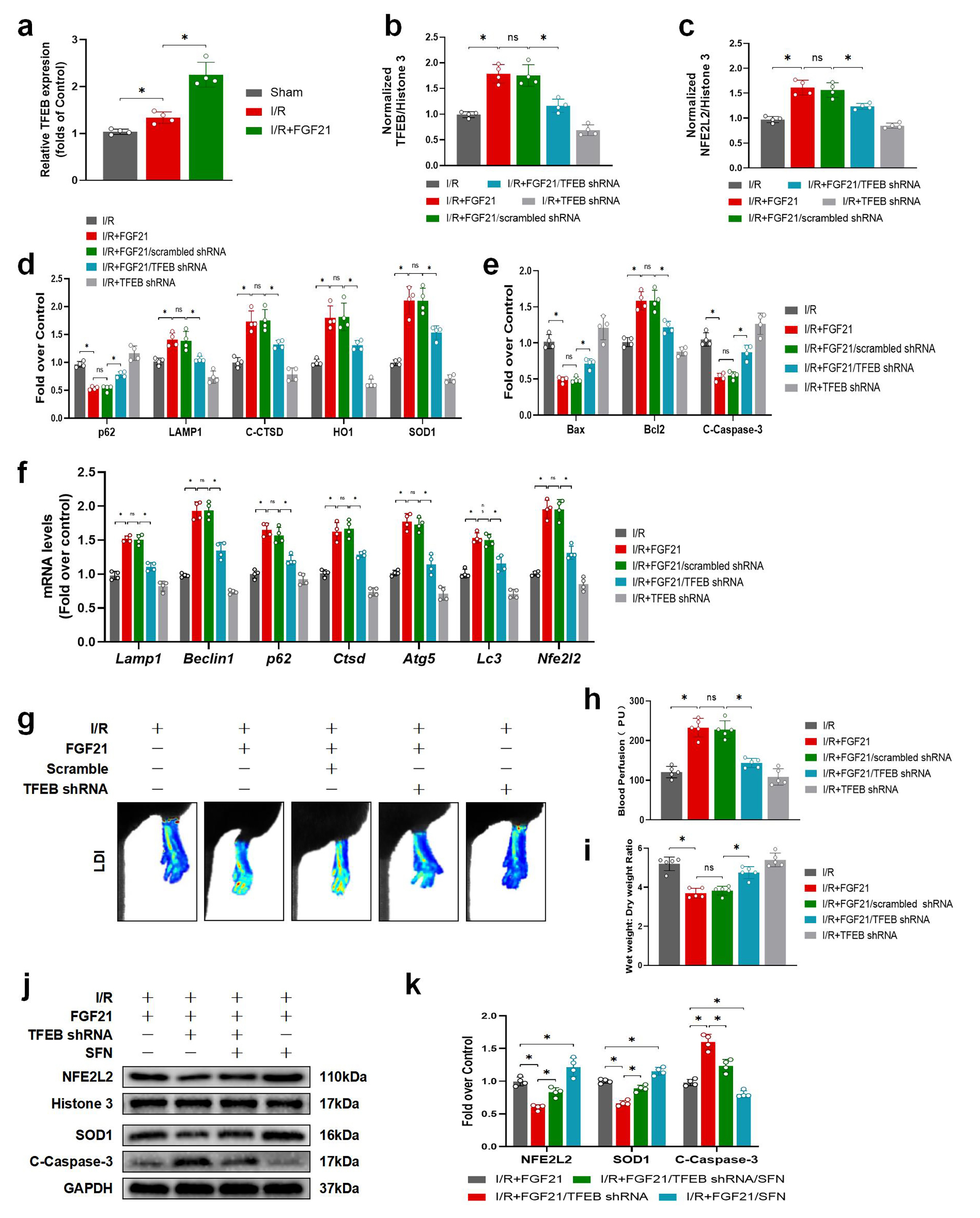
**

**Figure S9.** FGF21 restores autophagic flux, attenuates oxidative stress and reduces EC death via enhancing TFEB activity in I/R limbs. Mice were treated saline, FGF21, FGF21+scrambled shRNA, FGF21+TFEB shRNA and TFEB shRNA then underwent I/R. (**a**) Quantifcations data from (Fig. 1m) showing the protein level of TFEB. (**b-e**) Corresponding densitometric analysis of the bands from (Fig. 1p) normalized to the loading control. (**f**) Relative mRNA level of *Lamp1, Beclin1, p62, Ctsd, Atg5, Lc3* and *Nfe2l2* in skeletal muscle tissues of the indicated groups normalized to control β-actin. (**g**) Blood perfusion of hind limbs were detected by LDI. (**h**) Histogram showing signal intensity of blood ﬂow in I/R limbs. (**i**) Wet weight to dry weight ratio. (**j**) Western blots for oxidative stress and apoptosis markers. Mice were treated FGF21, FGF21+TFEB shRNA, FGF21+TFEB shRNA/SFN and FGF21+SFN then underwent I/R. (i) Wet weight to dry weight ratio. (**k**) Corresponding densitometric analysis of the bands from (j) normalized to the loading control. Data are expressed as the means ± SD (n = 4-5 per group). Significance: ns stands for not significant, ^*^*P* < 0.05.

**Figure S10**


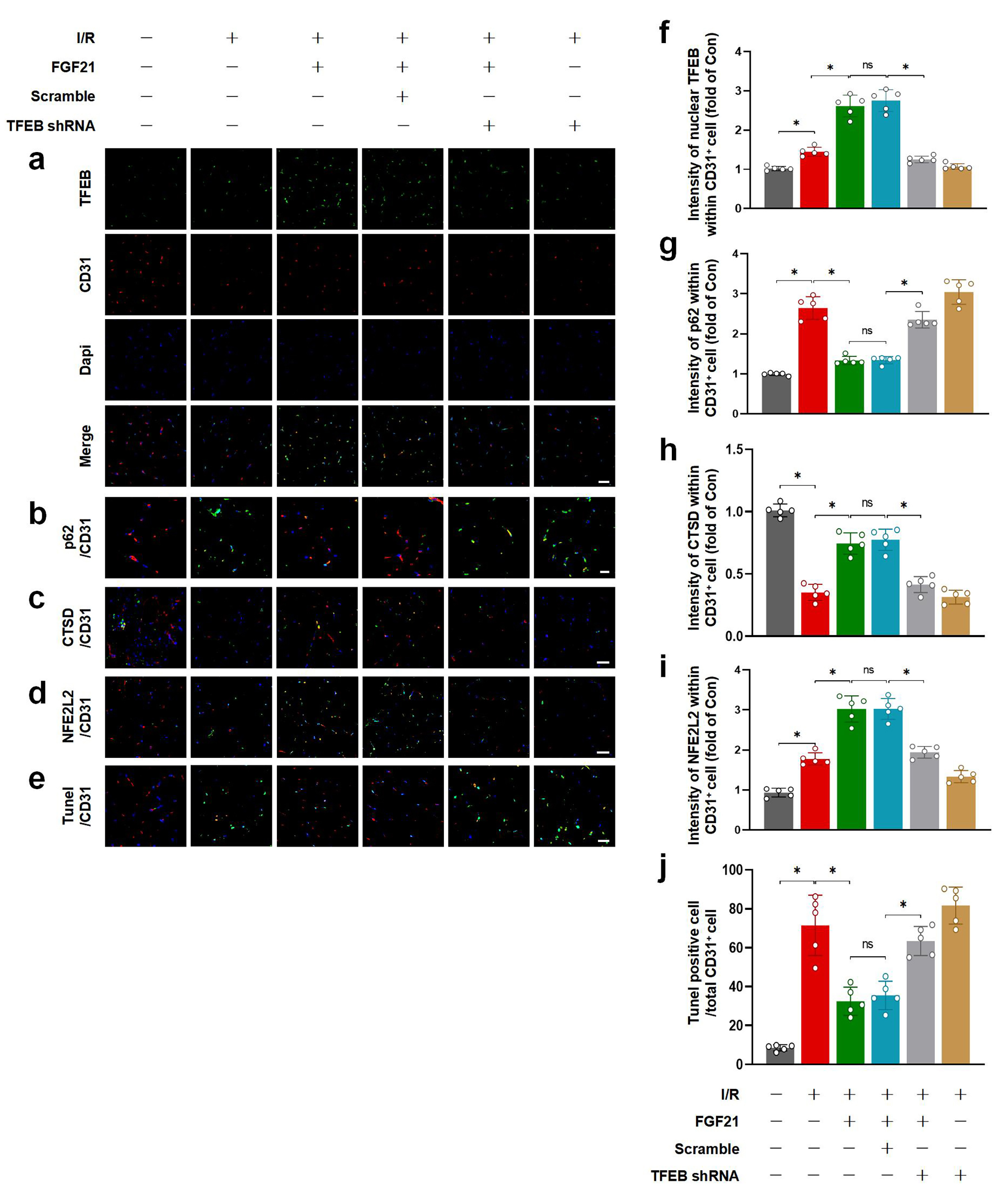


**Figure S10.** FGF21 restores autophagic flux, attenuates oxidative stress and reduces EC death via enhancing TFEB activity in I/R limbs. Mice were treated saline , FGF21, FGF21+scrambled shRNA, FGF21+TFEB shRNA and TFEB shRNA then underwent IR. (**a-e**) Images of skeletal muscle sections stained with antibodies against TFEB/CD31 (a, scale bars = 100 μm), p62/CD31 (b, scale bars = 50 μm), CTSD/CD31 (c, scale bars = 100 μm), NFE2L2/CD31 (d, scale bars = 100 μm) and TUNEL/CD31 (e, scale bars = 100 μm) , merged images include DAPI staining. (**f**) Quantification of immunofluorescence data from (a) showing the mean optical intensity of nuclear TFEB within CD31^+^ cell in the skeletal muscle. (**g-i**) Quantification of immunofluorescence data from (b-d) showing the mean optical density of p62 (b), CTSD (c) and NFE2L2 (d) on vascular endothelium (CD31^+^ cell) in the skeletal muscle. (**j**) Quantifcation of TUNEL and CD31 double-positive cells, the percentages of double positive cells versus total CD31 positive cells are indicated, data from (e). Data are expressed as the means ± SD (n = 5 per group). Significance: ns stands for not significant, ^*^*P* < 0.05.

**Figure S11**

**
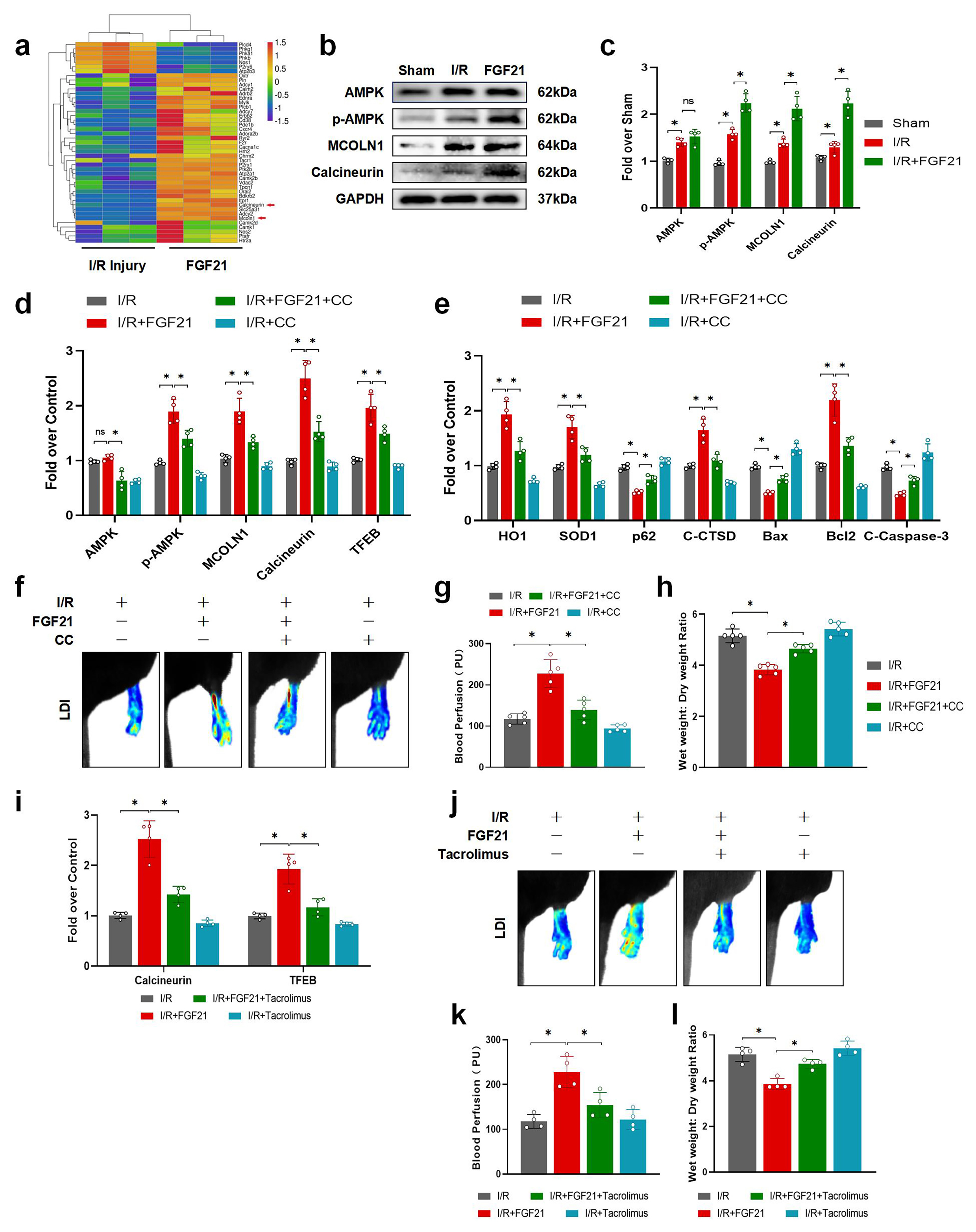
**

**Figure S11.** FGF21 activates TFEB via the AMPK-MCOLN1-calcineurin signaling pathway. (**a**) Heatmap displays DEGs of calcium signaling pathway regulated by FGF21. (**b**) Western blots for AMPK, p-AMPK, MCOLN1 and calcineurin in skeletal muscle tissues. Mice were administered with saline or FGF21 and subsequently underwent I/R. (**c**) Corresponding densitometric analysis of the bands from (b) normalized to the loading control GAPDH. (**d**) Quantifcations of protein levels of AMPK, p-AMPK, MCOLN1 and calcineurin with normalized to the loading control GAPDH and protein levels TFEB with normalized to the loading control Histone 3, data from (Fig. 1r). (**e**) Corresponding densitometric analysis of the bands from (Fig. 1s) normalized to the loading control GAPDH. (**f**) Blood perfusion of hind limbs were detected by LDI. Mice were treated saline, FGF21, FGF21+CC and CC then underwent IR. (**g**) Histogram showing signal intensity of blood ﬂow in I/R limbs. (**h**) Wet weight to dry weight ratio. (**i**) Quantifcations of protein levels of calcineurin with normalized to the loading control GAPDH; and protein levels TFEB with normalized to the loading control Histone-3, data from (Fig. 1t). (**j**) Blood perfusion of hind limbs were detected by LDI. Mice were treated saline, FGF21, FGF21+tacrolimus and tacrolimus then underwent IR. (**k**) Histogram showing signal intensity of blood ﬂow in I/R limbs. (**l**) Wet weight to dry weight ratio. Data are expressed as the means ± SD (n = 3-5 per group). Significance: ns stands for not significant, ^*^*P* < 0.05.
